# Impact of a pediatric infectious disease consultation service on timely step-down to oral antibiotic treatment for bone and joint infections

**DOI:** 10.1007/s15010-022-01934-4

**Published:** 2022-10-06

**Authors:** Katrin Mehler, André Oberthür, Ayla Yagdiran, Sarina Butzer, Norma Jung

**Affiliations:** 1 Department of Pediatrics, Faculty of Medicine, University Hospital of Cologne, Kerpenerstr. 62, 50397 Cologne, Germany; 2 Department of Orthopedics and Trauma Surgery, University Hospital of Cologne, Cologne, Germany; 3 Department I of Internal Medicine, University Hospital of Cologne, Cologne, Germany

**Keywords:** Children, Bacterial arthritis, Osteomyelitis, Discitis, Infectious diseases consultation service

## Abstract

**Purpose:**

In recent years an earlier step down to oral antibiotic therapy has been advocated for numerous infections. Trained infectious disease specialists regularly consulting their colleagues may speed up the implementation of such recommendations into clinical practice and thus may improve treatment.

**Methods:**

We retrospectively analyzed bone and joint infections in children admitted to the University Hospital of Cologne between 2010 and 2021. We assessed clinical, imaging, and microbiological findings and treatment modalities. Additionally, we assessed both the impact of a newly implemented pediatric infectious diseases consultation service and publications on revised treatment recommendations by comparing antibiotic therapy in two periods (2010–2016 versus 2017 to 2021).

**Results:**

In total, 29 children presented with osteomyelitis, 16 with bacterial arthritis and 7 with discitis. In period 2 (2017–2021) we observed shorter duration of intravenous treatment (*p* = 0.009) and a higher percentage of oral antibiotic treatment in relation to the total duration of antibiotics (25% versus 59%, *p* = 0.007) compared to period 1 (2010–2016).

Yet, no differences were identified for the total length of antibiotic treatment. Additionally, biopsies or synovial fluid samples were retrieved and cultured in more children in period 2 (*p* = 0.077). The main pathogen identified in osteomyelitis and bacterial arthritis was Staphylococcus aureus (MSSA), diagnosis was confirmed predominantly with MRI.

**Conclusion:**

Recent guidelines addressing the safety of an earlier step-down (to oral) antibiotic therapy have influenced clinical practice in the treatment of bone and joint infections in our hospital. A newly implemented pediatric infectious diseases consultation service might have accelerated this progress resulting in a faster step down to oral treatment.

## Introduction

Treatment recommendations for infectious diseases in children are frequently derived from clinical studies conducted in adults because high-quality studies in children are lacking. However, there are significant pathophysiologic differences between pediatric and adult bone and joint infections [1]. In children, bones and vertebral discs have blood supply via metaphyseal and periosteal vessels to the intravertebral discs. Consequently, many bone and joint infections in children are of hematogenous origin and antibiotics can easily penetrate the site of the infection. Therefore, it was assumed that shorter and early step-down to oral antibiotic treatment in pediatric bone and joint infections is feasible. In fact, evidence of the safety of shorter courses of antibiotics [2] and of a faster switch to the oral route [3] has grown significantly in the last 15 years and found entrance into current treatment recommendations [4–7]. Regular pediatric Infectious Diseases (ID) specialists counseling may accelerate the transition of new insights and recommendations regarding optimized antibiotic treatment into everyday practice. Numerous studies in adults have demonstrated an improved adherence to quality-of-care standards by regular ID consultations [8–10]. Reports on the effect of pediatric ID counseling are still limited but support an increasing demand [11] and an improvement of antibiotic treatment [12, 13].

In 2017, a pediatric infectious diseases consultation service was implemented in our tertiary care hospital. We hypothesized that the regular consultations on antibiotic treatment accelerated the transfer of concepts such as “shorter and less iv” into daily clinical practice. Taking this into account, we aimed to assess treatment strategies of pediatric bone and joint infections within a 12-year period, including 7 years (2010–2016) before and 5 years (2017–2021) after the implementation of our pediatric ID counseling service.

## Methods

The hospital database was searched for patients < 18 years admitted to the University of Cologne Children´s Hospital between January 2010 and December 2021 with diagnoses of bacterial arthritis, osteomyelitis, and discitis. The international classification of diseases, 10th revision was used for coding. The codes that were included were M00.0-, M00.1-, M00.2-, M00.8-, M00.9-, M46.- and M86.-. All charts of the children identified by the search were reviewed by a pediatric infectious disease’s specialist and an orthopedist with pediatric expertise. All imaging (Magnetic Resonance Imaging = MRI, Computer Tomography = CT, X-Ray and ultrasonography) had been conducted and/or assessed by a pediatric radiologist.

### Definitions

Diagnostic criteria for osteomyelitis were clinical features suggestive of a bone infection in combination with an imaging study with abnormalities characteristic of osteomyelitis (e.g., bone marrow inflammation or fluid collection adjacent to bone), elevated CRP values and an isolated pathogen from blood or biopsy. If no pathogen was identified, we used response to antimicrobial therapy to confirm the diagnosis. Infants with suspected osteomyelitis were included in the study if clinical and imaging studies showed typical findings AND if a pathogen was isolated OR in culture-negative cases if the patient responded to antimicrobial treatment.

Bacterial arthritis was confirmed in children with isolation of a bacterial pathogen from the synovial fluid. In culture-negative arthritis the combination of clinical signs (fever, joint pain and/or swelling), laboratory values (elevated synovial fluid WBC count showing > 50,000 cells/µl with > 90% of polymorphonuclear leucocytes) and/or radiographic findings (effusion) were used to corroborate the diagnosis.

Discitis typically occurs in young children and shows a gradual onset frequently without fever or distinctly elevated inflammation markers. MRI is the mainstay of diagnosis documenting involvement of the disc. Consequently, discitis was diagnosed in children with low grade clinical infection and demonstration of disc inflammation in MRI.

### Statistical analysis

Data analyses were performed using SPSS 27.0 (IBM, Munich, Germany). Hypotheses in the univariate analysis were evaluated with Mann–Whitney *U* test for continuous variables. A *p* value of < 0.05 was considered statistically significant. We used a uniform dataset with available data for all metric parameters.

## Results

In total, 87 children diagnosed with osteomyelitis, dicitis or bacterial arthritis were retrieved from the hospital database. After evaluation 33 children were identified not to fulfill the criteria for these infections. Instead, the majority had cellulitis (*n* = 8) or reactive arthritis (*n* = 6). Of note, 9 children coded for osteomelitis actually had chronic nonbacterial osteomyelitis (recurrent multifocal osteomyelitis CRMO). The remaining 10 had trauma (*n* = 2), osteoid osteoma (*n* = 2) and one child each had leukemia, vitamin D deficiency, rheumatoid arthritis and acute disseminated encephalomyelitis. In two children, the diagnosis remained unclear.

The diagnosis of a bone or joint infection was confirmed in 54 children, 30 suffered from (infective) osteomyelitis, 16 from bacterial arthritis and 8 from discitis. We excluded two patients with chronic infection due to tuberculous osteomyelitis and Brucella discitis from further analysis because they were not considered appropriate for a comparison on the role of oral versus intravenous antibiotic treatment due the length of standard therapy.

Baseline characteristics of the patients are given in Table 1. Children with discitis rarely presented with fever and had lower CRP values on admission compared to children with osteomyelitis and bacterial arthritis. Children with osteomyelitis and bacterial arthritis predominantly presented with pain and fever, children with bacterial arthritis often had additional joint swelling.

**Table 1:** Baseline patient´s characteristics and findings on admission

	Osteomyelitis, *n* = 29	Bacterial arthritis, *n* = 16	Discitis, *n* = 7
Age (y)	8 [3–10]	8 [1–12]	1 [1, 2]
Latency to admission (d)	5 [2–14]	4 [1–6]	21 [9–31]
Male	20 (69%)	12 (75%)	5 (71%)
Antibiotic treatment before admission	10 (35%)	3 (19%)	2 (29%)
CRP on admission (mg/l)	61 [4–139]	84 [30–118]	8 [2–17]
Fever on admission	16 (55%)	11 (69%)	1 (14%)
Major clinical findings	Pain + fever (15 (52%))	Pain + fever ± swelling (11 (69%))	Pain ± refusal to walk (4 (57%))
Predisposing factors*	8 (28%)	0 (0%)	0

Median [IQR], *n* (%) *sickle cell anemia, immunodeficiency, trauma, surgery, *M. perthes*

Detailed information on imaging, microbiological results and surgical procedures is provided in Table 2. The overwhelming pathogen identified in both osteomyelitis and bacterial arthritis was Methicillin susceptible *Staphylococcus aureus* (MSSA). The causative pathogens for the respective diseases and the sites these pathoges were obtained from are presented in detail in Table 2.

**Table 2:** Imaging, microbiology, and surgical procedures

	Osteomyelitis, *n* = 29	Bacterial arthritis, *n* = 16	Discitis, *n* = 7
Imaging			
Ultrasonographic findings	2/14* Fluid collection 2/14* Thickening of periosteum 1/14* Cortical defect	13/15* Effusion	0
MRI done and confirming diagnosis	27/29 (93%)	12/16 (75%)	7/7 (100%)
Microbiology			
Blood culture	9/27* MSSA 1/27* MRSA 1/27* *Candida albicans* 1/27* *Kingella kingae*	5/15* MSSA	0 none positive
Synovial fluid, intraoperative biopsy): culture	6/14* MSSA 2/14* MRSA 1/14* *Streptococcus pyogenes* 1/14* *Haemophilus influenza*/*Streptococcus intermedius* 1/14* *Salmonella gloucester* 1/14* MSSA, *Pseudomonas aeruginosa, Finegoldia magna*	6/16 MSSA 1/16 *Streptococcus agalactiae*	0
Synovial fluid/intraoperative biopsy: PCR	2/5* MSSA	1/6* *Streptococcus pneumoniae*	0
Surgical procedures			
Time from admission to surgery (d)	3 [1–5]	1 [0–5]	No surgery
Needle aspiration	7 (23%)	15 (94%)	0
Arthrotomy	0	1 (6%)	0
Abscess drainage	4 (14%)	0	0
Other**	3 (10%)	0	0

*Total stated due to missing numbers (ultrasound/ culture/PCR not done), **debridement, removal of plate/screws, Methicillin susceptible *Staphylococcus aureus* (MSSA); Methicillin resistant *Staphylococcus aureus* (MRSA)

In most patients, the diagnosis was made or confirmed by MRI. In children with bacterial arthritis effusions were frequently identified by ultrasound and suggestive of the diagnosis though 75% had an additional MRI for confirmation. In contrast, in children with osteomyelitis, only one in three patients had ultrasonographic findings suggestive of the correct diagnosis such as fluid collection adjacent to the bone or thickening of the periosteum. Two children with osteomyelitis had indwelling foreign material, screws, and a wire cerclage, respectively, from previous surgery. As expected, ultrasonography was not able to identify discitis. Of note, all children with discitis received ultrasonography but not of the spine. The knees or hips were examined because the children regularly refused to walk or presented with a limp.

Blood cultures were obtained in > 90% of children with osteomyelitis, bacterial arthritis, and discitis. Positivity rates were 43%, 33% and 0, respectively. MSSA was the main pathogen that was isolated. Of note, in children who received antibiotic treatment before blood cultures were assessed the positivity rate was 36% (5/14) compared to 34% (12/35) without prior antibiotics.

One of the two children with osteomyelitis and all children with bacterial arthritis received surgical procedures. In children with bacterial arthritis, needle aspiration combined with irrigation of the joint was performed to identify the causative pathogen and to clear bacterial load. In children with osteomyelitis, the primary surgical procedures were needle aspiration and/ or abscess drainage.

Most children received at least two weeks of intravenous therapy followed by oral antibiotic treatment for a median of 10 days in children with bacterial arthritis, more than three weeks for osteomyelitis and 4 weeks for discitis. For initial intravenous treatment (empiric treatment), a combination therapy was chosen in over 60% of children with osteomyelitis and bacterial arthritis and in 100% of children with discitis. For oral treatment, most children received monotherapy. In period 2, we observed a trend to less combination therapy for both intravenous (58% versus 81%, *p* = 0.132) and oral treatment (4% versus 21%, *p* = 0.132),

In total, a variety of antibiotic substances and combinations were used for intravenous or oral treatment for the respective diagnosis. In patients where the causative pathogen was identified, initial treatment was effective in all but one child who presented with *Kingella kingae* osteomyelitis.

**Fig. 1:**
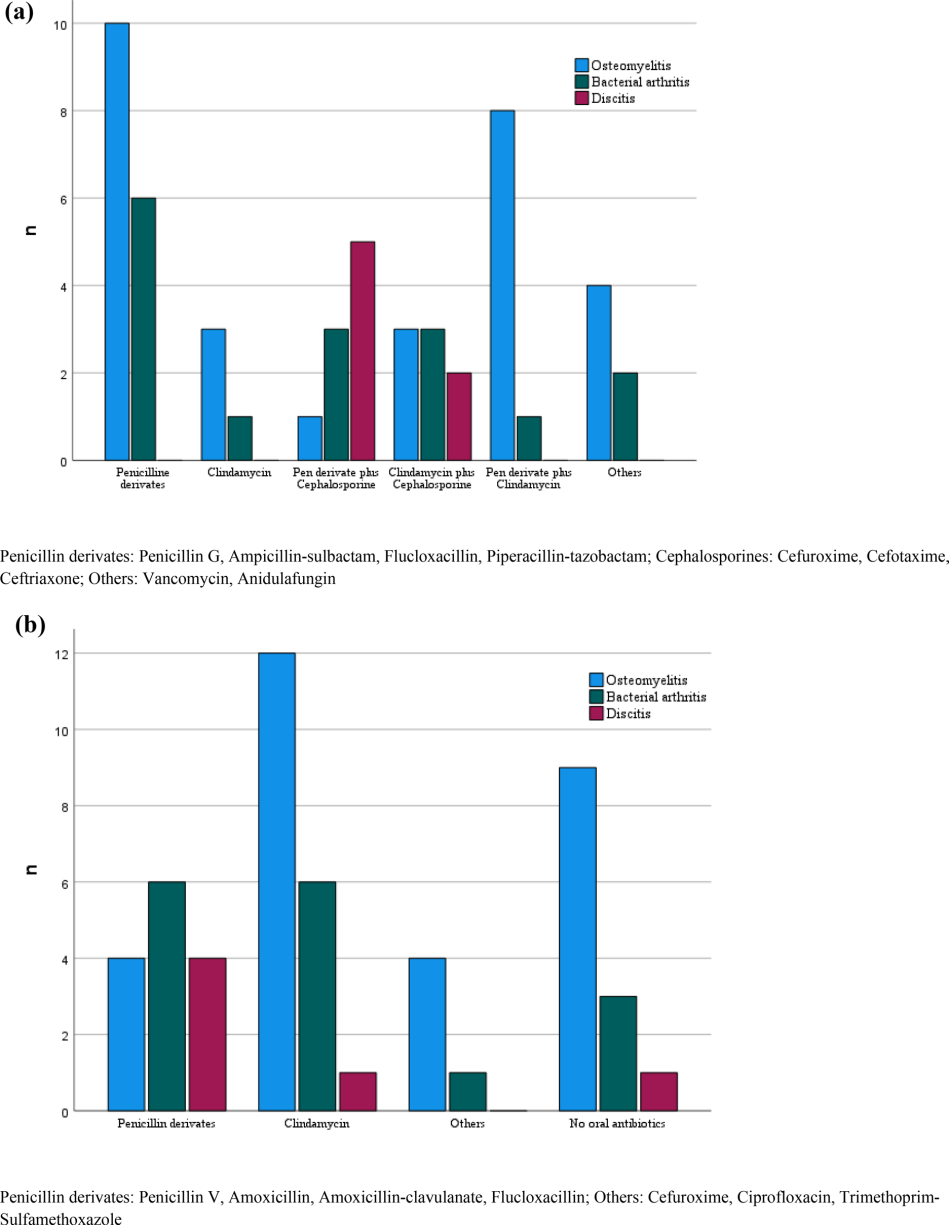
**a** Intravenous, **b** oral antibiotic treatment predominantly used for children with osteomyelitis, bacterial arthritis, and discitis

An overview illustrating the antibiotic classes that were most frequently applied is presented in Fig. 1a and 1b.

**Fig. 2:**
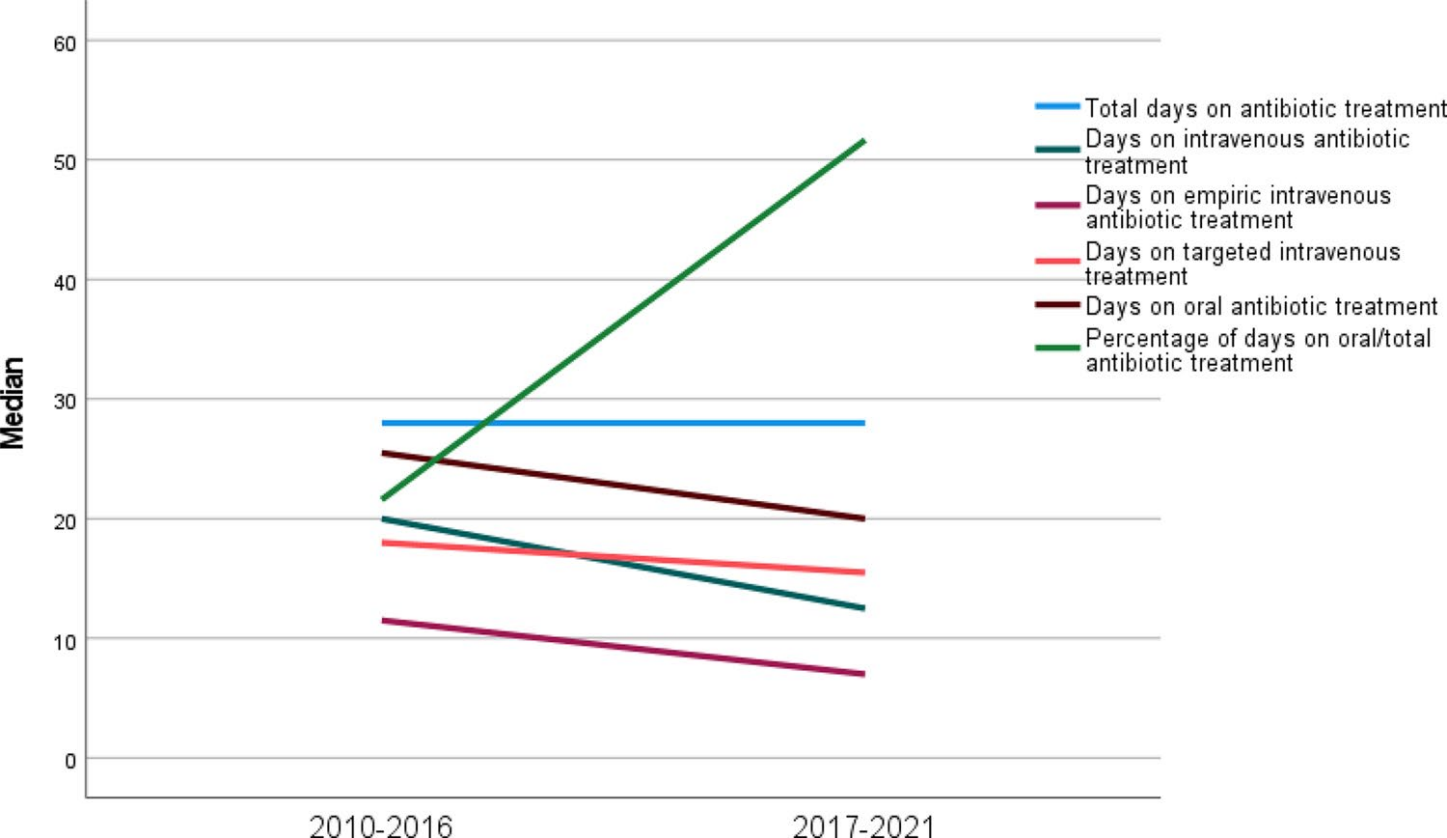
Median duration and application of antibiotic treatment in period 1 and 2

**Fig. 3:**
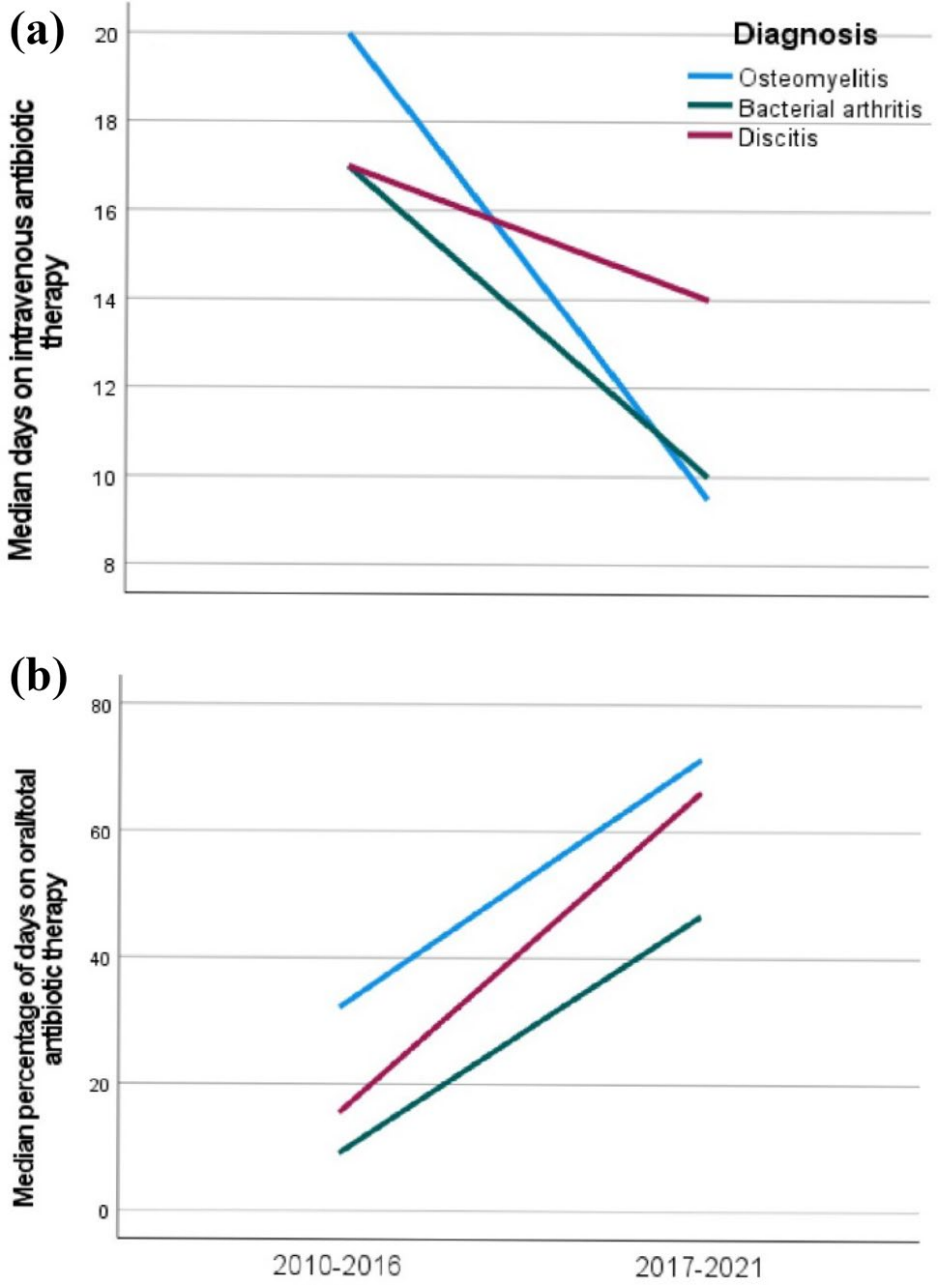
**a**, **b** Changes in duration of days on intravenous antibiotic treatment (**a**) and percentages of days on oral compared total days on antibiotic therapy (**b**) over both periods of time for osteomyelitis, bacterial arthritis, and discitis

To identify the effect of current guidelines and the start of training pediatricians in infectious diseases on the choice of therapy, we compared antibiotic treatment strategies in two periods (2010–2016 and 2017–2021). From 2010 to 2016, counseling by an infectious disease specialist (not specialized in pediatric infectious diseases) was requested in 10/26 (45%) children with bone and joint infections compared to 22/26 (85%) of children admitted from 2017 to 2021 who received a pediatric infectious disease counseling. Although no short-term relapses were reported, one child with MSSA osteomyelitis was readmitted 13 months after the first episode. The MRI demonstrated a Brodie’s abscess. Cultures from surgical specimen grew MSSA. Duration and application of antibiotic treatment are presented in Fig. 2. From 2017 to 2021, children had fewer days on intravenous antibiotics compared to 2010–2016 (*p* = 0.009). This is predominantly due to a decrease in empiric therapy. Furthermore, the percentage of oral days to total days of antibiotic therapy increased significantly from 25% in period 1 to 59% in period 2 (*p* = 0.007) (Fig. 3).

The changes of antibiotic treatment for the respective diagnoses are presented in Fig. 2. Of note, there was no difference in time from admission to surgery which was a median of two days for both periods. In period 2, more cultures from synovial fluids or biopsies were assessed (13/26 (50%) in period 1 versus 19/26 in period 2 (73%), *p* = 0.077). In contrast, there was no difference in the number of blood cultures (25/26 children had blood cultures in period 1 versus 24/26 in period 2). Furthermore, there was a trend to an earlier discharge from hospital in period 2 (Median days in hospital in period 1 20 days (IQR 12–23), in period 2 13 days (8–24), *p* = 0.206).

## Discussion

We present a comprehensive retrospective analysis of bone infections in children focusing on antibiotic treatment strategies comparing two time periods after publication of new guidelines and implementation of a pediatric infectious diseases’ consultation service.

Our study demonstrated an earlier switch to oral treatment of pediatric bone and joint infections in our hospital in the second period.

In children evidence of positive effects following the implementation of an infectious disease’s consultation service is still scarce. A German study assessed a pediatric ID counseling service as a part of a more extensive antibiotic stewardship bundle and demonstrated an improvement of antibiotic treatment [12]. Furthermore, an increasing demand for pediatric ID counseling was reported [11]. Consequently, it can be expected that, analogous to the extensive data from the adult population, a pediatric ID counseling improves adherence to quality-of-care antibiotic treatment strategies.

Compared to intravenous treatment oral antibiotic therapy reduces hospital durations, costs and complications associated with intravenous catheters [14]. Initiatives such as the “oral is the new i.v.” [15] or the “shorter is better movement” [16] promote earlier transition to oral and shorter duration of antibiotic treatment for numerous infections. In children, two randomized trials demonstrated the safety of shorter treatment for osteomyelitis (20 versus 30 days) [2] and bacterial arthritis (10 versus 30 days) [17]. In both studies a short phase of intravenous treatment (2–4 days) was followed by oral therapy. Furthermore, favorable outcomes were reported in a selected group of children who received oral treatment only [18]. Additionally, experts strongly recommend an early switch to oral for children with bacteremia who are treated with clindamycin or a first-generation cephalosporin and report good outcomes [19]. RCTs addressing an earlier switch to oral are still scarce and it is unlikely that large scales studies for pediatric patients will be realized. In this light, the review by McMullen that suggested general principles and recommendation on a timely and safe switch to oral antibiotic in children is an important tool to aid clinicians in their decisions [5].

For the families of children requiring antibiotic therapy, the switch to oral treatment generally facilitates discharge from hospital. Treatment at home offers numerous advantages for the affected families: The parents can go back to work, and the child may resume educational training and spare time activities[20]. Furthermore, painful, and stressful events due to the insertion of intravenous catheters and complications associated with indwelling intravenous catheters are reduced.

In our cohort, a variety of different antibiotics as well as a various combinations were used for initial treatment. Although the rate of combination therapy was declining over the years, still one in two children were initially treated with more than one antibiotic in period 2. The recommendations for empirical treatment differ widely in European countries. In low-risk settings most suggest monotherapy with an antistaphylococcal Penicillin, Clindamycin or a 1st- or 2nd-generation cephalosporin. Combination therapies are usually limited to infants < 3 months [6]. The current standard of care for pediatric bone and joint infections in our hospital is in line with the guidelines published by the ESPID in 2017 [6] and predominantly recommends monotherapy and to limit intravenous treatment to a few days. In relation to both aspects, our ID service has failed to implement any significant changes so far. Adherence to the guideline is only growing slowly and antibiotic treatment is frequently initiated at admission following personal preferences of the attending pediatrician. Additionally, despite growing contrary evidence, there is still a great concern for treatment failure associated with an early switch to oral treatment.

Our study is limited by its retrospective design. Therefore, follow-up data to evaluate the outcome are limited to readmissions to the same hospital. Relapses in children who were not hospitalized or admitted to a different hospital might have been missed. Additionally, we assessed data from one single center and may have missed potential confounders or unknown center-specific effects. Furthermore, we included all patients with clinical and radiographically confirmed bone and joint infections in our analysis and did not exclude those without a confirmed pathogen provided that the patient responded to antibiotic treatment. This definition was also applied to patients who received drainage although the clinical improvement could be due to drainage of the inflammatory region. This approach was chosen, because one in three patients had prior antibiotic treatment and routine PCR for pathogen identification had not been implemented. Additionally, no differences in outcomes were detected in patients with and without positive cultures and both were suggested to be treated similarly [21]. Finally, synovial fluid WBS counts were not obtained routinely. Consequently, children with contaminated samples could have been included. Nonetheless, due to the meticulous chart review by several experts, it is highly probable that the presentation, diagnosis and treatment of pediatric bone and joint infections as well as changes over the years were assessed and interpreted correctly. However, it is not possible to determine which factor (implementation of a pediatric infectious disease team, publication of new treatment guidelines, a combination of both or unknown further potentially explanatory factors) contributed the most to the changes in treatment concepts.

## Conclusion

Recent guidelines on pediatric bone and joint infections promote a timely switch from intravenous to oral antibiotics. These recommendations gradually find their way into clinical practice. Pediatricians who are specialized in infectious disease and regularly offer consultations for their colleagues may speed up this transfer.
